# Learning by moving: Physical motion accelerates category learning

**DOI:** 10.1126/sciadv.aed4003

**Published:** 2026-10-23

**Authors:** Luke Rosedahl, Yuka Sasaki, Takeo Watanabe

**Affiliations:** ^1^ Department of Biomedical Engineering, Florida Atlantic University, Boca Raton, FL, USA.; ^2^ I-SENSE, Florida Atlantic University, Boca Raton, FL, USA.; ^3^ Cognitive and Psychological Sciences, Brown University, Providence, RI, USA.

## Abstract

Categorization is crucial for survival, helping animals forage, navigate, and avoid predators—all tasks involving movement. Yet, dominant theories of category learning treat it as a purely cognitive or perceptual process. Here, we challenge this view by demonstrating that physical movement can enhance category learning—but selectively. Using immersive virtual reality, we found that performing large actions, such as swinging a lightsaber, substantially improved learning of categories requiring integration of multiple cues (e.g., distinguishing aggressive from noncancerous lesions), while having minimal impact on categories involving independent evaluation of features (e.g., sorting by color and shape). This selective enhancement reveals a previously unrecognized motor contribution to categorization and may explain why prior studies missed it, opening avenues for optimizing training in critical categorization tasks.

## INTRODUCTION

Category learning is fundamental to many everyday decisions such as how much to slow down on a road (icy or wet) and expert performance in domains such as radiology. In real-world contexts, categorization is embedded in action and requires coordinated perceptual-motor responses (e.g., deciding whether a fruit is ripe and picking it). Yet, decades of research have viewed category learning primarily as a high-level cognitive or perceptual function that is independent from the physical action involved in expressing the categorization decision. For example, exemplar theory (*1*, *2*) and prototype theory (*3*, *4*) (two of the most dominant theories of category learning) assume that categorization occurs by comparing the current object to abstract mental representations, either stored individual examples (exemplar theory) or a generalized average (prototype theory). In both cases, the theories treat the motor response as purely output, occurring after the categorization decision and playing no role in the learning process itself. This disconnect between theory and the embodied nature of real-world decisions raises a fundamental question: Does how we move during categorization shape how we learn?

Outside of category learning, a substantial body of work has found that response movement influences learning and performance. Motor response has been shown to improve interception performance (*5*), facilitate visual letter recognition (*6*, *7*), bias visual feature selection during visual search (*8*), and enhance perceptual sensitivity to action-relevant features (*9*). Unexpectedly, despite frequent references to how categories guide behavior (*10*–*12*), no prior work has examined how the sensorimotor dynamics of the response influence category learning. One previous study that included conditions that varied the required motor response [button press on a keyboard versus arm movement and button press in virtual reality (VR)] found no difference in learning between the conditions, although this study was not explicitly looking for a role of motor engagement in learning and did not include within-modality comparisons of movement (within-VR button versus arm movement) (*13*).

One reason motor engagement may have appeared irrelevant in prior studies is the type of category structure used. Many studies [including the previously mentioned study (*13*)] use category structures that can be solved by combining binary decisions across features (e.g., if it has whiskers and meows, it is a cat). These structures, often called rule-based (RB) categories (Fig. 1, D and E), can be learned through explicit, verbalizable rules and are thought to depend primarily on prefrontal systems involved in hypothesis testing and working memory, neural systems that are likely less sensitive to motor dynamics (*10*, *11*, *14*).

**Fig. 1.**
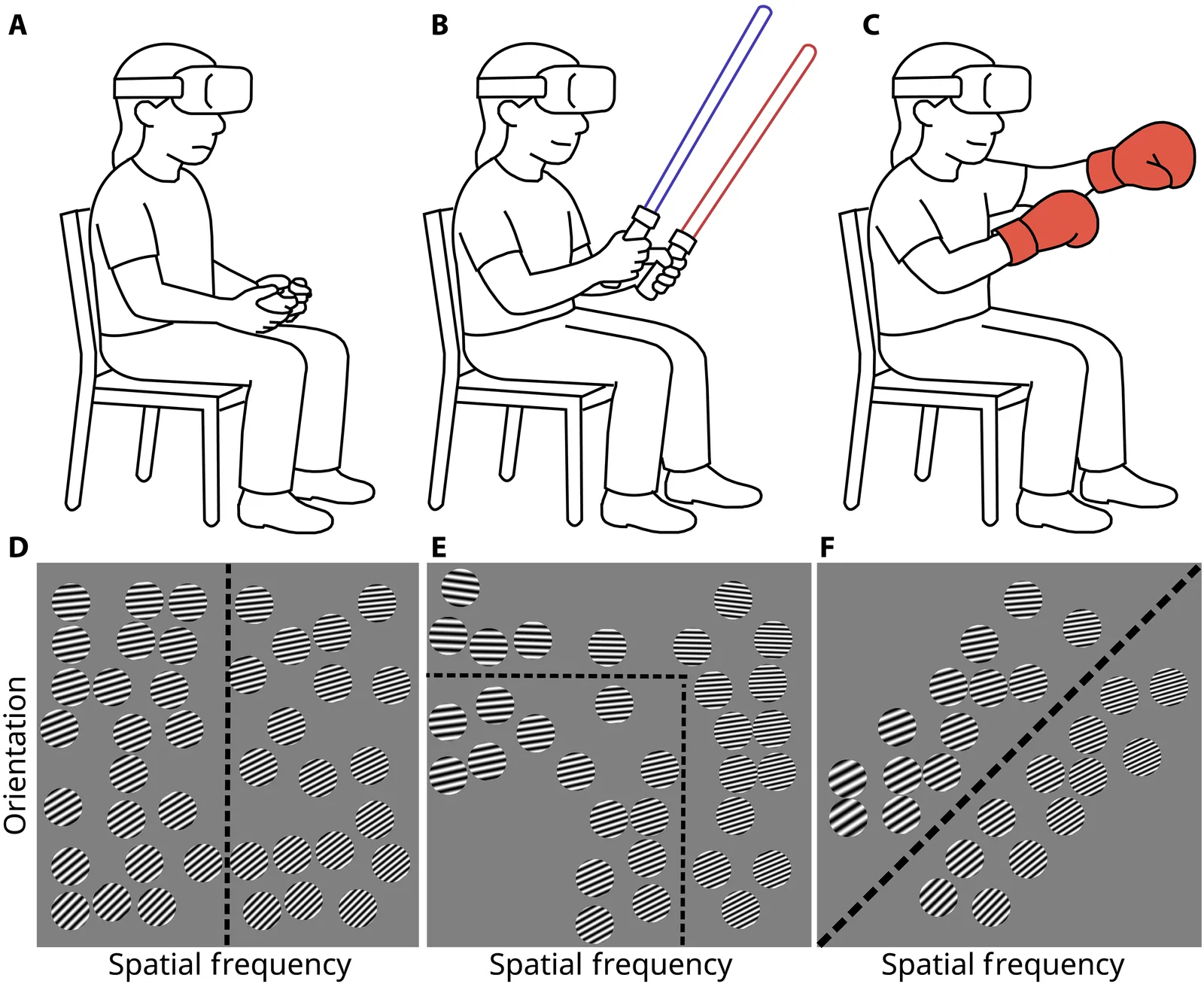
Response conditions and category structures. (**A** to **C**) The figures show diagrams of the response conditions for participants in the (A) button press, (B) lightsaber, and (C) punch conditions. Figures in (**D**) to (**F**) show examples of (D) one-dimensional RB, (E) conjunctive RB, and (F) II category structures. The structures are shown with example stimuli (striped discs) that vary in the spatial frequency (thickness) on the *x* axis and orientation (angle) on the *y* axis. The dashed line represents the category boundary.

However, not all category structures can be solved through explicit rules. Another common type of category structure (often called Information-Integration or II) requires combining information across dimensions before making a decision (e.g., a clinician’s sense of whether a lesion looks aggressive, integrating many features without being able to articulate the exact rule). II structures (Fig. 1F) are often learned in a similarity-based fashion by comparing the current object to previously experienced ones (*15*–*17*) and are thought to rely on procedural learning systems including the striatum and associated motor circuits (*11*, *14*). Notably, no prior study has manipulated motor response magnitude for II structures.

In this study, we tested whether motor response influences not only RB but also II structures. To this end, we developed an immersive VR paradigm that allowed systematic manipulation of response type: finger presses (Fig. 1A) and full arm movements that either interacted with the stimulus (lightsaber swing to hit the stimulus; Fig. 1B) or did not interact with the stimulus (punching a response box; Fig. 1C). Consistent with the previous study, learning of RB categories was largely unaffected by movement (with only a slight, inconsistent, and nonsignificant decrease by full-arm movements). In contrast, learning for similarity-based II categories was dramatically improved by full-arm movements, regardless of whether the movement involved direct interaction with the stimulus. This selective effect indicates that the benefit for similarity-based categories arises from the role of movement itself in category learning, not from broader motivation or engagement, since greater engagement would have improved performance for both category types.

These results suggest that motor responses can influence category learning performance rather than serving only as a response output. This provides evidence to challenge the long-standing assumption that motor movement is simply the output of category learning. Instead, our findings reveal that the dynamics of response movement may be integrated into learning, motivating a reexamination of category learning theories and suggesting directions for improving training paradigms across a wide range of challenging real-world tasks, from medical diagnostics to human-AI interactions.

## RESULTS

### Swinging speeds up learning for similarity-based tasks

In experiment 1, participants (*N* = 80; mean age = 21.4 years, SD = 5.2; 57.5% female) learned to categorize stripes of different angles and thicknesses for one of two category structures (RB or II; see Fig. 1, D and F for schematic and Materials and Methods for full details) while responding by either pulling the trigger or hitting the stimulus with a lightsaber (button or swing). Participants completed 600 trials split into 12 blocks and were allowed to take a self-timed break between blocks if desired.

Performance was analyzed across blocks using a series of nested generalized linear mixed models (GLMMs) with main effects for category structure (RB versus II), response type (button versus swing), and block along with random subject-level effects for intercept and learning rate. Model selection was performed using AIC, which balances goodness of fit against model complexity to favor models with better predictive performance; equivalent analyses using BIC, which penalizes complexity more strongly than AIC, supported the same primary conclusions (Supplementary Materials). We complemented this analysis with bootstrap significance testing examining the difference between response conditions across blocks to identify precisely when performance differences emerged between response conditions. All primary inferential claims rest on the GLMM analyses; the block-wise bootstrap comparisons are descriptive and shown to illustrate the temporal pattern of differences across training. Per-block significance markers are reported without multiple-comparisons correction. These should be interpreted on the basis of the overall pattern across blocks: Sustained clusters of consecutive significant differences are more suggestive of a consistent difference than isolated single-block markers, which are consistent with chance variation under uncorrected testing.

We found that swinging significantly enhanced learning for II categories (Fig. 2A) but had a minimal negative effect on RB categories (Fig. 2B). See fig. S1 for individual learning curves. Our best-fitting GLMM showed a significant interaction between response type and category structure [β = 0.42, SE = 0.15, *P* = 0.005, odds ratio (OR) = 1.52], indicating that learning for the category structures was affected differently by the response type. To explore this in more detail, we performed GLMMs separately for both the II and RB category structures. For the II structure, the GLMM revealed a significant main effect of response type (β = 0.27, SE = 0.11, *P* = 0.01, OR = 1.31), with swinging response type improving performance. For the RB structure, the GLMM found an insignificant effect of response type (β = −0.10, SE = 0.08, *P* = 0.25, OR = 0.90), with swinging slightly decreasing performance and the best fitting GLMM including no effect of response type. See tables S1 and S2 for all model fitting results from this section.

**Fig. 2.**
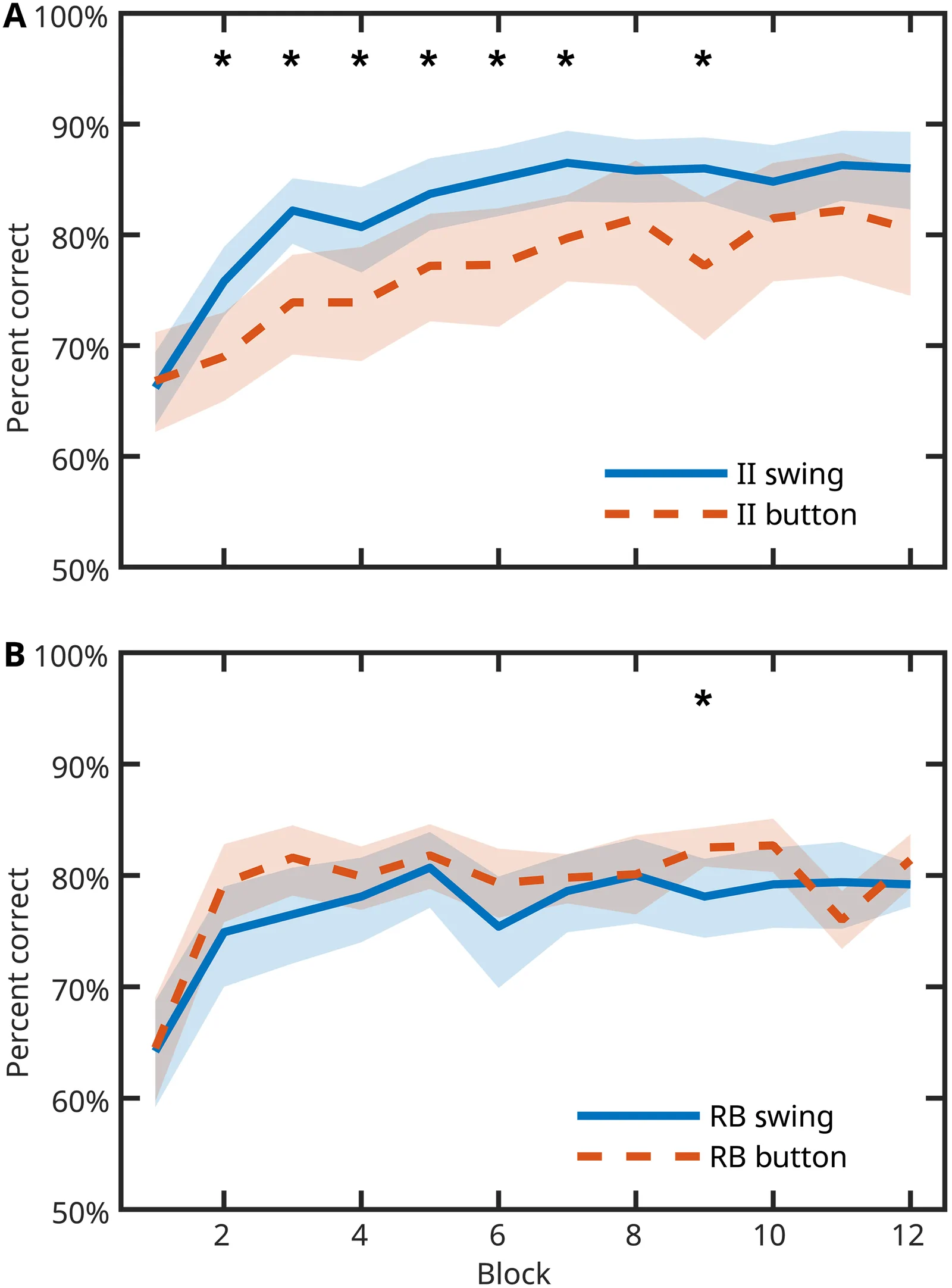
Experiment 1 learning performance. Learning curves showing percent correct for (**A**) II and (**B**) RB category tasks for swing (solid blue) and button (dashed orange) conditions. Shaded regions represent 95% bootstrap confidence intervals (CIs). Asterisks indicate descriptive uncorrected block-wise comparisons (*P* < 0.05; uncorrected).

To determine when performance differences emerged, we examined the descriptive bootstrap analysis that revealed sustained advantage for swinging for the II structure at blocks 2 to 7 and 9 (uncorrected *P*s < 0.05; Fig. 2A) and continued (although nonsignificant) advantage through the end of learning. This early and sustained advantage suggests that larger motor responses facilitated II category learning from early in training. For the RB structure, the bootstrapping analysis showed a difference only at a single isolated block (block 9; Fig. 2B) with button outperforming swing, further suggesting that response modality had minimal impact on RB category learning.

These findings suggest that swinging responses differentially affect category learning depending on the task type, with substantial benefits for II categories but minimal impact for RB categories. However, there are several alternative explanations for this result that do not include a benefit from movement, including the possibility that direct interaction with the stimulus or longer stimulus exposure in the swing condition drove the improved performance. In addition, the simplicity of our RB category structure might have obscured potential effects of response modality on RB learning. To confirm that this was not the case, we performed experiment 2 to address these alternative explanations.

### Punching increases learning and final performance for similarity-based categories

Experiment 1 showed a clear benefit of movement for similarity-based (II) categories and no benefit for RB categories. However, several nonmovement factors could explain this effect: interaction with the stimulus during the response; longer stimulus presentation times due to slower reaction times (RTs) in movement conditions; the possibility that participants in the movement condition took more time to make decisions, which could have improved performance [consistent with the speed-accuracy tradeoff (*18*)]; and a ceiling effect for the relatively simple RB categories (which may have allowed near-perfect performance regardless of movement). In addition, it is possible that movement aids retrieval rather than encoding. That is, movement might not help participants form better category representations during training, but instead larger movements at decision-time may directly facilitate access to those representations. Under this account, switching to a smaller movement response should degrade performance if the movement benefit depends on response-specific retrieval support.

To rule out these accounts and replicate the initial finding, we conducted experiment 2 (*N* = 80; mean age = 21.0 years, SD = 5.3; 65.0% female). Participants either punched a response box in front of them or pulled the controller trigger. Punching required a larger motor response that did not interact with the visual stimulus, directly testing the stimulus-interaction concern. Each stimulus was presented for 2.5 s independent of response time, equating visual exposure. RT was recorded on every trial and analyzed to evaluate speed-accuracy explanations. To rule out ceiling effects for the RB condition, the RB category was made more complex (Fig. 1E for schematic and Materials and Methods for full details), requiring rule discovery on each stimulus dimension. Last, a 100-trial response-modality switch block was included after training to test whether benefits reflect response-specific retrieval.

### Movement benefit is not due to stimulus interaction or stimulus presentation time

Comparing punching (no stimulus contact) with trigger-pulling showed that punching produced a robust advantage for II categories, improving both learning rate and final performance (Fig. 3A). See fig. S2 for individual learning curves. A GLMM for the II conditions confirmed a significant interaction between block and response type (β = 0.06, SE = 0.02, *P* = 0.01) and a trend toward a main effect of response type (β = 0.24, SE = 0.13, *P* = 0.08, OR = 1.27), indicating that the movement benefit was not driven by stimulus interaction. See tables S3 and S4 for all model fitting results from this section. The significant interaction term indicates that punching increased learning rate, with the performance enhancement emerging across training rather than appearing as a uniform boost. Because stimulus presentation time was equated across conditions, this also confirms that the benefit from movement is not due to differences in presentation time.

**Fig. 3.**
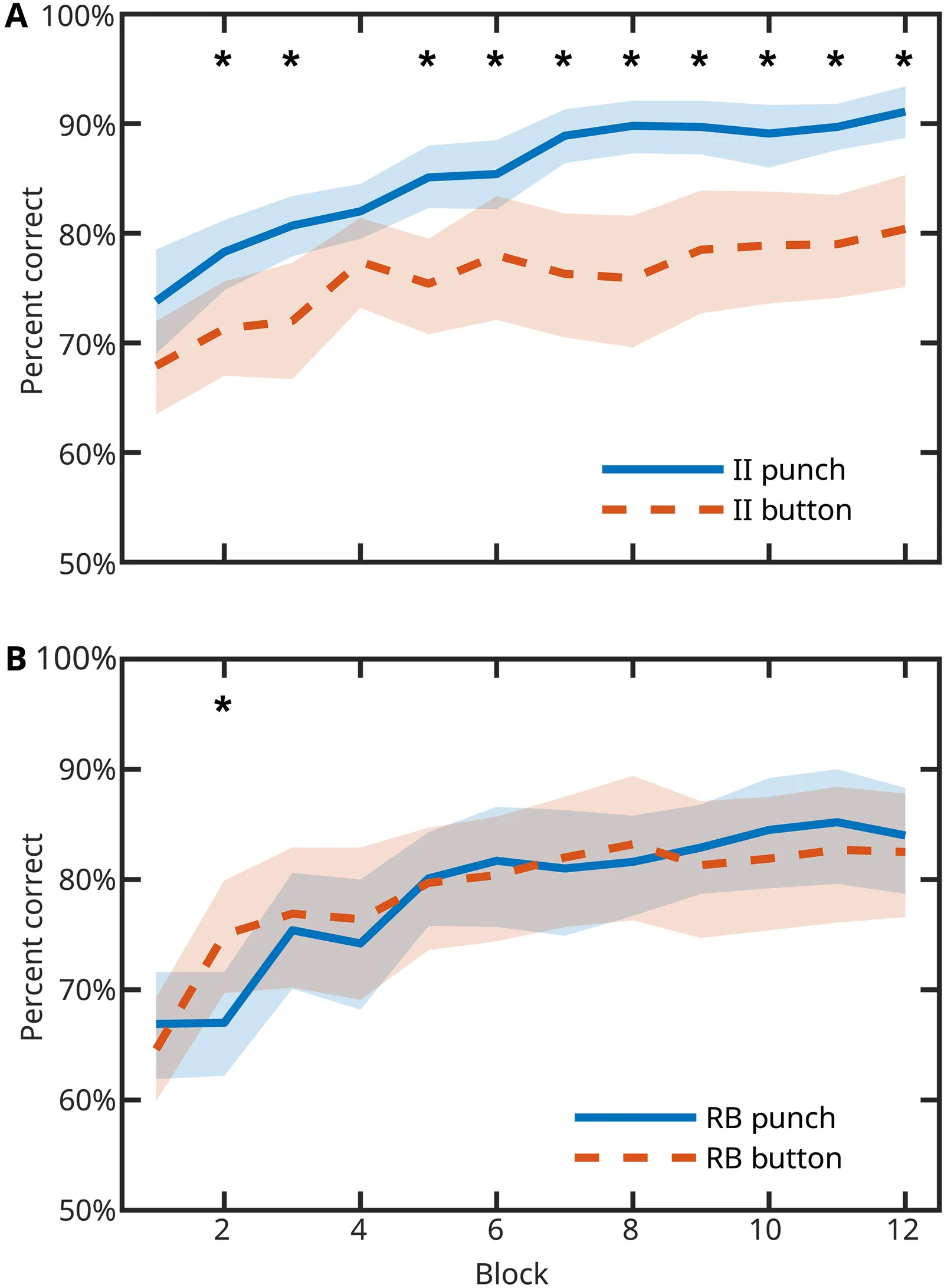
Experiment 2 learning performance. Learning curves showing percent correct for (**A**) II and (**B**) RB category tasks for punch (solid blue) and button (dashed orange) conditions. Shaded regions represent 95% bootstrap CIs. Asterisks indicate descriptive uncorrected block-wise comparisons (*P* < 0.05; uncorrected).

### Movement benefit is not explained by RT (speed-accuracy tradeoff)

Because slower responses can sometimes improve accuracy, we directly examined RT. Even after accounting for RT, punching significantly improved II performance. For an intuitive check, trials were binned by RT across participants and accuracy compared within each bin; if speed differences drove the effect, accuracies would converge across conditions, yet II punching remained superior (with the exception of sparsely populated tail bins; Fig. 4A). The primary analysis of trial-wise GLMMs with nested models (full model: main effects of response type, trial, and RT; all two-way interactions; random intercepts for subjects; random slopes for trial and RT) selected the full model as best fitting. Critically, after accounting for RT the GLMM showed a main effect of response type (β = 0.83, SE = 0.29, *P* ≤ 0.005) and an interaction of response type × trial (β = 0.001, SE = 0.0005, *P* = 0.006). RT coefficients were negative across models (slower RT → worse performance), the opposite of a speed-accuracy account. Thus, RT differences do not explain the movement advantage. Beyond addressing speed-accuracy concerns, the convergence of this trial-wise GLMM with the block-wise analyses reported above indicates that our main conclusions are stable across reasonable analytic choices. See table S5 for all model fitting results including RT.

**Fig. 4.**
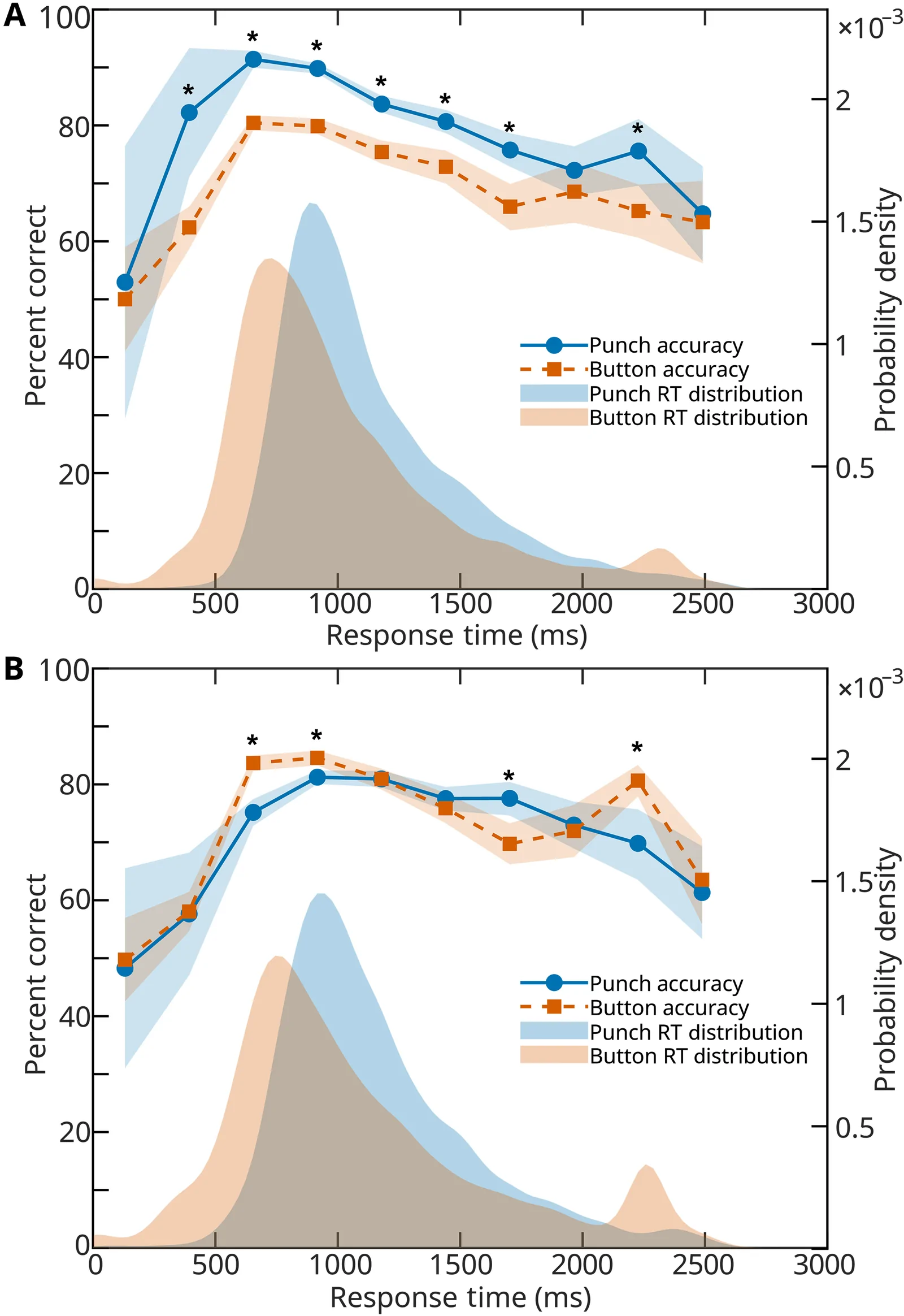
Response accuracy versus response time. (**A**) II category structure and (**B**) conjunctive RB category structure. Top lines show performance across RT bins for punch (blue solid line) and button responses (orange dashed line). The shaded regions represent 95% bootstrap CIs, with asterisks indicating bins with statistically significant differences (*P* < 0.05 uncorrected). Response time distributions for each condition are shown as shaded areas at the bottom of each panel.

### No ceiling-effect explanation for RB categories

For the more complex RB structure, punching produced only a slight, insignificant negative effect early in learning (block 2 only; Fig. 3B). The best-fitting GLMM included only block, and a model including response type showed an insignificant negative response-type effect (β = −0.13, SE = 0.15, *P* = 0.39, OR = 0.88). Hence, the lack of movement benefit for RB learning is not due to ceiling performance.

### Movement benefit is not limited to response-specific retrieval

If movement helped only response-specific retrieval, switching response modality at transfer should eliminate (or reverse) the advantage. Instead, II participants trained with punching outperformed the button-trained group both at the end of training (Fig. 5; punch training: *M* = 0.907, 95% confidence interval (CI) [0.889, 0.924]; button training: *M* = 0.810, 95% CI [0.766, 0.851]; bootstrapped t test: *P* < 0.00001, Cohen’s *d* = 1.30) and during transfer (punch → button: *M* = 0.895, 95% CI [0.878, 0.912]; button → punch: M = 0.831, 95% CI [0.794, 0.863]; bootstrapped *t* test: *P* < 0.0005, Cohen’s *d* = 1.05). The punch-trained group showed only minimal performance loss when switching to button at transfer (from *M* = 0.907 to *M* = 0.895). A retrieval account in which decision-time movement drives the advantage would predict substantial degradation. Instead, the advantage was preserved nearly intact, indicating that the benefit was established during training and does not require the trained response to be available at test.

**Fig. 5.**
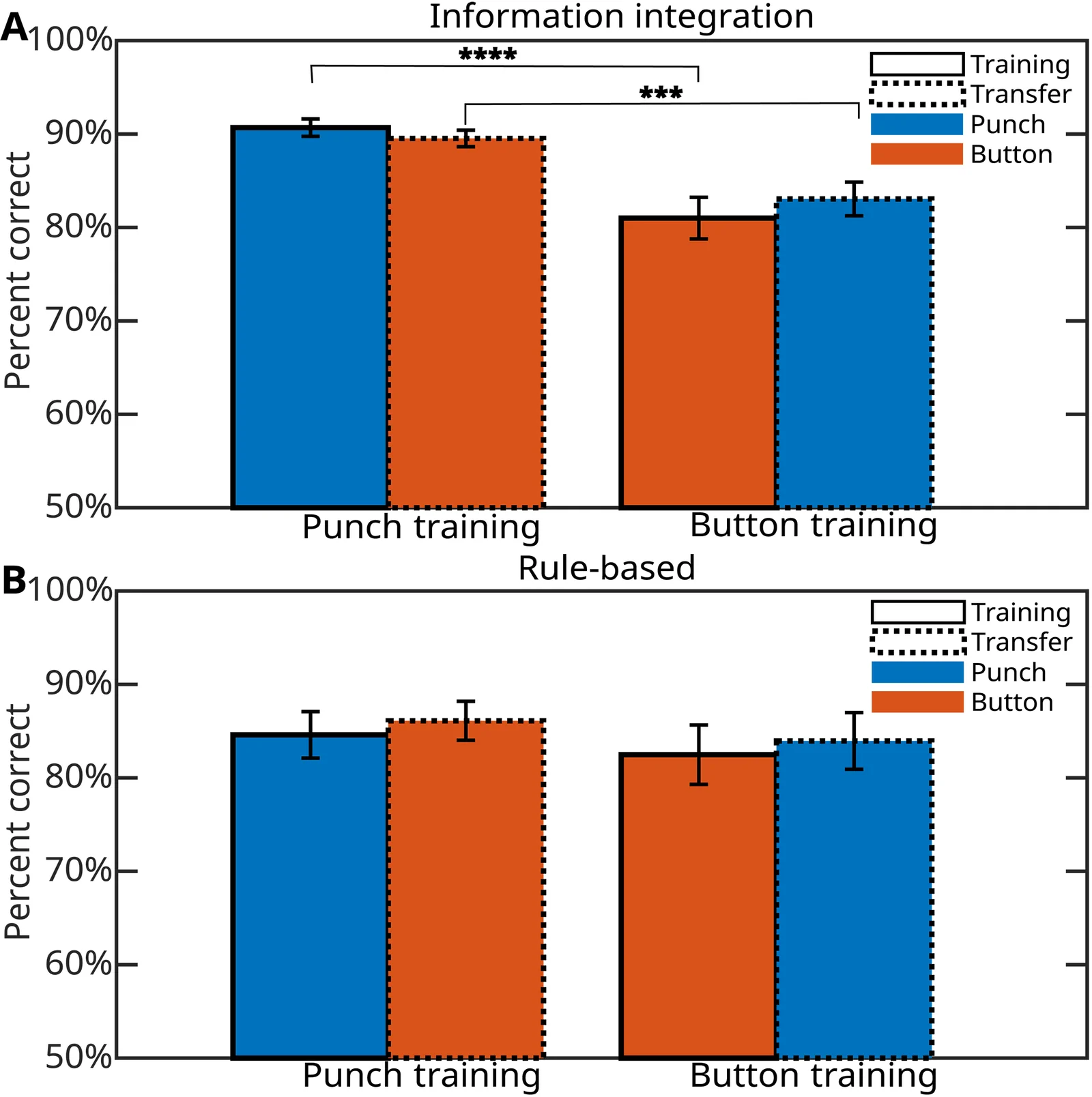
Learning transfer across modalities. Performance (% correct) during the last 100 trials of training (solid outline) and transfer block using the opposite response mode (dotted outline) for II (**A**) and RB (**B**) category learning. Error bars represent SE. Significance markers are: ***P < 0.001 and ****P < 0.0001.

### Overall interaction of response type and category structure

Across the full dataset, a GLMM revealed a significant response-type × category-structure interaction (β = 0.51, SE = 0.19, *P* = 0.008, OR = 1.66), replicating and strengthening experiment 1: Movement selectively benefits II learning, with minimal negative impact on RB learning.

## DISCUSSION

The finding that larger movements provide a dramatic benefit for category learning, at least for II categories, challenges traditionally held views of category learning that view it as a purely cognitive and perceptual process. Many cognitive theories (such as exemplar theory and prototype theory) model category learning as the process of mapping between representation and category, ending the learning pipeline before movement is factored in and therefore being unable to capture the results reported here. Even the highly influential neurobiologically detailed theory Competition between Verbal and Implicit Systems (COVIS) (*14*), which includes pathways mapping from category representations to motor cortex regions (*19*), ignores the type of movement itself. The results reported here suggest that while these theories can capture certain aspects of learning, to fully understand category learning, we must consider the dynamics of motor responses as well. This is especially crucial because, unlike laboratory experiments, many of the real-world category learning tasks we seek to understand require motor responses beyond simple button presses.

Why would larger movement help learning for II categories but not RB categories? Broadly speaking, this selective pattern is consistent with the different neural systems these tasks are thought to rely on: II learning is thought to depend on procedural systems closely tied to motor circuitry, while RB learning is suggested to depend more on prefrontal hypothesis-testing systems. While the results presented here cannot isolate the mechanism of this effect, the selective pattern we observe is consistent with the behavioral consequences of this proposed link and points to several possible mechanisms. One possibility is that larger movements increase the amount of learning-induced plasticity in the striatum (a brain region heavily involved in motor control and motor learning). Larger motor movement costs have been found to increase striatal dopamine release (*20*–*22*) and firing rates (*23*–*25*), which could increase learning-related plasticity. In addition, movement cost may also influence attentional allocation. Higher-effort actions to access information have been shown to guide attention toward task-relevant features (*26*, *27*). Response movement cost could play a similar role, potentially influencing learning in tasks that require feature integration. However, future research is needed to test these possibilities.

Regardless of the underlying mechanism, the results reported here cast light on another long-debated issue within the field of category learning: the relatively slow learning and prevalence of suboptimal strategies for II categories. Decades of work have found that compared to RB categories, II categories tend to be learned slower (*28*) and are more affected by experimental factors such as feedback delay (*29*), switching the mapping between responses and categories (*19*), and the trained visual field location (*30*, *31*). All these studies used button press responses, which, on the basis of the results reported here, could have been constraining the learning systems responsible for these tasks. While it is unlikely that all previously found differences between these two tasks were driven by suboptimal motor responses in the experimental paradigms, this motivates a careful reexamination of previous findings to ensure that they were not affected by suboptimal motor response.

Looking ahead, it will be important to explore whether movement effects generalize to other category structures and whether motor-related individual differences affect learning. For example, rule-plus-exception (*32*) and prototype distortion (*33*) tasks may engage different neural systems and rely on distinct learning strategies. These systems may interact with motor processes in unique ways, potentially leading to different movement-related effects. Similarly, there are individual differences in motor preferences (such as handedness and previous experience with motor tasks), which were not recorded here and could alter the extent to which movement benefits learning. Understanding how movement influences learning across a broader taxonomy of category structures and how it interacts with individual motor characteristics could help refine existing models and clarify when motor engagement enhances or interferes with learning.

This work contributes to growing evidence that category learning interacts with neural processes beyond those traditionally considered by the field. For example, previous work has shown that category learning can rely on early visual processing, showing location specificity that is a hallmark of those visual areas (*30*, *31*). Combined with this work showing that category learning also interacts with response output dynamics, this suggests that there are substantial benefits to examining category learning through an integrated viewpoint that considers how all stages of sensory processing, decision making, and response output affect category learning.

In addition, our results may be relevant to the field of embodied cognition, which studies how movement affects task performance (*34*). Unlike many embodied cognition findings where gestures contain meaning relevant to the task at hand [such as using hand movements to represent spatial relationships (*35*)], our results suggest that movement-related benefits can occur even when the movement is not semantically related to the task. The dissociation between the RB and II tasks may also help frame some of the inconsistencies seen in the embodied cognition literature, where similar task designs can lead to differential impact of movement on task performance (*36*–*38*). One speculative possibility is that small differences in task design alter the cognitive or neural systems engaged by the task, resulting in disparate outcomes despite seemingly consistent tasks.

These insights also raise important questions about populations with long-term motor restrictions due to injury or aging (*39*). Reduced movement due to mobility impairments has been shown to affect tactile perception (*40*) and is linked to general cognitive decline (*41*). The results reported here suggest that these populations may also exhibit reduced performance in category learning tasks that require integrating across multiple features. This would represent an overlooked but crucial effect, which could be an important target for rehabilitation programs.

These findings challenge how immersive VR paradigms for learning are typically designed. While immersive VR environments enable a wide range of motor responses, many existing paradigms do not incorporate larger, effortful movements, often defaulting to button presses to mimic task-performance settings that were designed to minimize movement costs. Our results suggest that this design choice may overlook a critical opportunity: strategically engaging motor systems during learning before transitioning to lower-movement conditions to reduce fatigue and maintain efficiency during task execution. This insight opens a previously unexploited direction for immersive VR paradigm design: to leverage motor engagement not just for realism or interaction but also as a mechanism for optimizing cognitive and neural processes underlying learning.

By showing that larger movements can enhance performance in a controlled VR paradigm, our results raise the possibility that motor engagement may be relevant for future training approaches. Tasks that require integrating across multiple features are often learned through extended periods of practice, and previous work has shown that providing instructions on how to perform these types of tasks provides little to no benefit (*42*–*45*). Adding a larger motor component to the tasks may therefore be worth testing as a potential way to improve training efficiency while also providing a rich behavioral signal (movement dynamics) that may offer a window into the internal state of the learner. Overall, the present findings motivate future research testing whether larger motor movements during training can improve learning in challenging real-world domains such as radiology, aviation, and education.

## MATERIALS AND METHODS

### Participants

One hundred sixty participants (mean age = 21.2 years, SD = 5.3; 61.3% female) were recruited from the area surrounding Brown University (20 per condition; *n* = 80 in experiment 1; *n* = 80 in experiment 2). Twenty participants per condition was planned on the basis of previous studies using these category structures. All participants had normal or corrected-to-normal vision and participants received either $10 or course credit for participating. The institutional review board at Brown University approved the study protocol (IRB# 1203000582), and all participants provided written informed consent.

### VR setup

Participants wore an HTC Vive Pro 2 headset and remained seated throughout the experiment. All participant interaction with the experiment was done through the Vive Pro 2 controllers. The virtual environment consisted of a long hallway with neutral lighting. The stimuli would appear in front of the participant, and they would respond by either pulling a trigger on one of the two controllers, hitting the stimulus with a virtual lightsaber or punching a response box in front of them. The experiment was developed in Unity (version 2019.2.12f1) using the SteamVR SDK. In the swing and punch conditions, responses were registered via collision-based hit detection using Unity’s physics engine. Response mappings were randomized by handing participants the controllers in no particular order. Handedness was not recorded or counterbalanced, which is a limitation of the current design. This approach is common in category learning studies, although future work will consider more systematic control of motor-response mapping.

### Stimuli and categories

In all conditions and experiments, the categorization stimuli were grayscale gratings presented in a VR environment. Each grating appeared as a circular disc with varying spatial frequency (thickness of lines) and orientation (angle of lines). Gratings were presented at 1 unit of distance from the participant at a size of 5° visual angle and were centered in a larger gray disc of size 20° visual angle. For each experiment and condition, the category structures were generated in arbitrary 0 to 100 space. For both experiments, the II category structure was that used previously in computer-based experiments (*16*, *42*). This structure was created by randomly sampling stimuli from two uniform distributions with length = 50 units, width = 10 units, and a between-category separation of 10 units across a diagonal boundary with slope of 1 (Fig. 6A). For experiment 1, the RB category was that used in (*16*) for the long near condition. The categories were created by generating an II category structure in a similar manner to the one just described but with a between-category spacing of 0 and then rotating the structure 45° about the midpoint (Fig. 6B). For experiment 2, the RB category was that used in (*42*). The categories were defined by a right-angle boundary in the stimulus space with two segments: (75, 45) to (75, 75) and (45, 75) to (75, 75). Stimuli were randomly drawn from a uniform distribution over the area and assigned into one of the two categories depending on whether they were within or outside of the angle and whether they fell within 4 to 14 units of the boundaries (Fig. 6C).

**Fig. 6.**
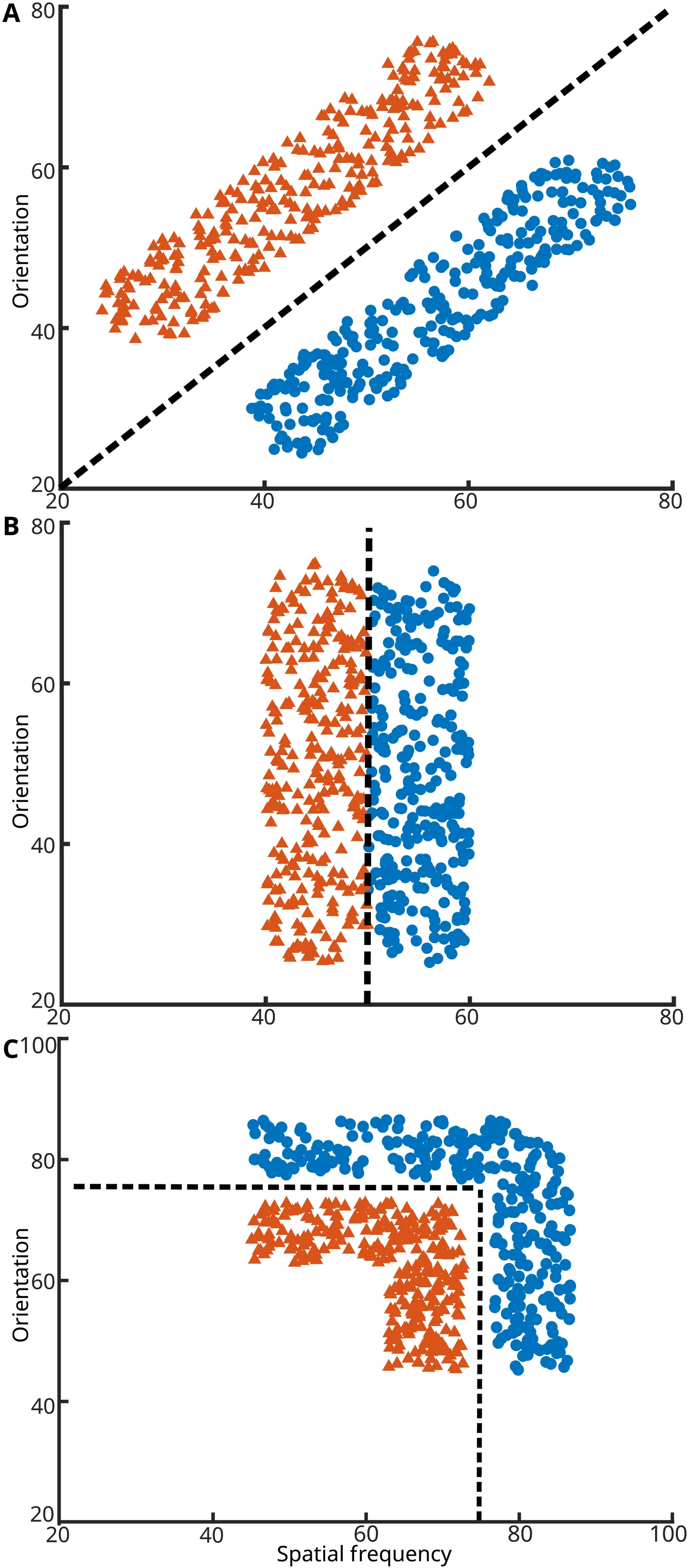
Category structures. Category structures used for the experiments 1 and 2 II condition (**A**), experiment 1 RB condition (**B**), and experiment 2 RB condition (**C**). Orange triangles represent one category, blue circles represent the other, and the dashed line is the optimal category boundary.

For all structures, the values were then converted to orientation and spatial frequency using the same formulas as the previous papers using these category structures: Spatial frequency *x*∗ = [*x*/30 + 0.25] cycles per degree visual angle and orientation *y*∗ = [0.9*y* +20] degrees counterclockwise from the horizontal. The order of the stimuli was randomized across participants.

### Procedure: Experiment 1

Participants (*N* = 80) were randomly assigned to one of four conditions in a 2 × 2 between-subjects design crossing category structure (RB versus II) with response type (button versus swing). In the button condition, participants responded by pressing the trigger button on one of the two controllers. In the swing condition, participants responded by hitting the stimulus with one of two virtual lightsabers, which were rendered as (0.05, 0.05, 1.0) unit cubes starting at the end of the controllers. In both conditions, the lightsabers were present; however, in the button condition, nothing happened in response to the stimuli getting hit by the lightsabers.

Participants completed 600 trials of category learning with response feedback split into blocks of 50 trials. Participants were instructed to categorize the stripes into one of two categories by either pulling the trigger on the left or right controller (button condition) or hitting the stimulus with the left or right lightsaber (swing condition). Participants were not provided with any information on the category structures or possible strategies. On each trial, the stimulus remained until the subject made a response (response-terminated), at which point the stripes would turn green if the subjects were correct or red if the subjects were incorrect. After changing color, the disc would start moving toward the participant at a rate of 1 unit per second until it disappeared 0.5 units in front of the participant (0.5-s feedback display time). There was then a 0.5-s break before the next stimulus appeared. Participants were encouraged to take self-paced breaks between blocks if desired but to not pause during blocks.

### Procedure: Experiment 2

Experiment 2 (*N* = 80) used the same 2 × 2 design crossing category structure (RB versus II) with response type (button versus punch). In the button condition, participants responded by pressing the trigger button on one of the two controllers. In the punch condition, participants responded by punching a response box (1, 0.5, 0.1) units in size, which was located 0.5 units in front of them. Each controller was rendered with a red cube (0.1, 0.1, 0.1) to represent a boxing glove.

Unlike experiment 1, stimulus timing was set in experiment 2. Each stimulus would appear, remain stationary for 2 s, and then begin moving at a rate of 1 unit per second until disappearing 0.5 units in front of the participant (total stimulus presentation time 2.5 s). As soon as the participant responded, the area around the stripes would turn green or red to signify the correctness of their response, but the stripes would remain visible and trial timing would continue to follow the set timeline. After each stimulus disappeared, there was a 0.5-s break before the next stimulus appeared. If participants did not respond to a stimulus before it disappeared, the same stimulus was shown again to ensure that all participants categorized all stimuli. For two participants, the experiment code did not show the stimulus again if they did not respond in time, resulting in 1 and 10 missed trials, respectively, from the first two participants of the II punch condition. These missed trials are treated as chance performance.

Participants completed 600 training trials with their main assigned response modality, split into 50-trial blocks. Participants were instructed to categorize the stripes into one of two categories by either pulling the trigger on the left or right controller (button condition) or punching the response box with the left or right controller (punch condition). Participants were not provided with any information on the category structures or possible strategies. As in experiment 1, participants were encouraged to take self-paced breaks between blocks if desired but not to pause during a block. After completing 600 trials, participants were then informed through written instructions in the VR environment that they would switch response modality (e.g., from punching to button). They were informed that the categories would remain the same, so if they would have punched with the left controller they should pull the left trigger. This transfer phase used the same category structure, stimuli, timing, and response feedback as the training phase. Three participants (one each from the II punch, RB punch, and II button conditions) were unable to start the transfer stage because of timing constraints but completed the main training. Their data are included in primary analysis but are not reflected in the transfer analysis.

### Data analysis

Category learning performance was measured as the proportion of correct responses in each block. All analyses were conducted using MATLAB R2021a (MathWorks, Natick, MA). Learning curves were analyzed using GLMMs with a logit link function, binomial count number correct for each block, and Laplace fitting all implemented using MATLAB’s fitglme function. The full models included fixed effects for category structure (RB, II), response type (button, swing/punch), block number, and their interactions, as well as random intercepts and slopes for individual participants. For experiment 2, we analyzed the relationship between response time and accuracy by binning trials based on response time. We then compared across conditions for the same response time bin using bootstrapped significance testing with replacement and 100,000 iterations. This was supported by additional trial-wise GLMM analysis including response time to determine whether movement had an impact on learning after controlling for response time. For all GLMMs, the model with the best AIC was selected as the best fitting model, although selecting on the basis of BIC returned results supporting the main conclusions as well. All pairwise *t* test comparisons were performed using bootstrapping with replacement for 100,000 iterations.
