# Supplementary material for: Learning by moving: Physical motion accelerates category learning
Source: Sci Adv. 2026 Oct 23;12(43):eaed4003. doi: 10.1126/sciadv.aed4003 (PMC13693652; doi:10.1126/sciadv.aed4003)
Supplement: Supplementary file 1 [file sciadv.aed4003_sm.pdf]

Science Advances
AAAS

Supplementary Materials for

Learning by moving: Physical motion accelerates category learning

Luke Rosedahl *et al.*

Corresponding author: Luke Rosedahl, lrosedahl@fau.edu

Sci. Adv. **12**, eaed4003 (2026)
DOI: 10.1126/sciadv.aed4003

This PDF file includes:

Figs. S1 and S2
Tables S1 to S5

Fig. S1: Individual Learning Curves from Experiment 1

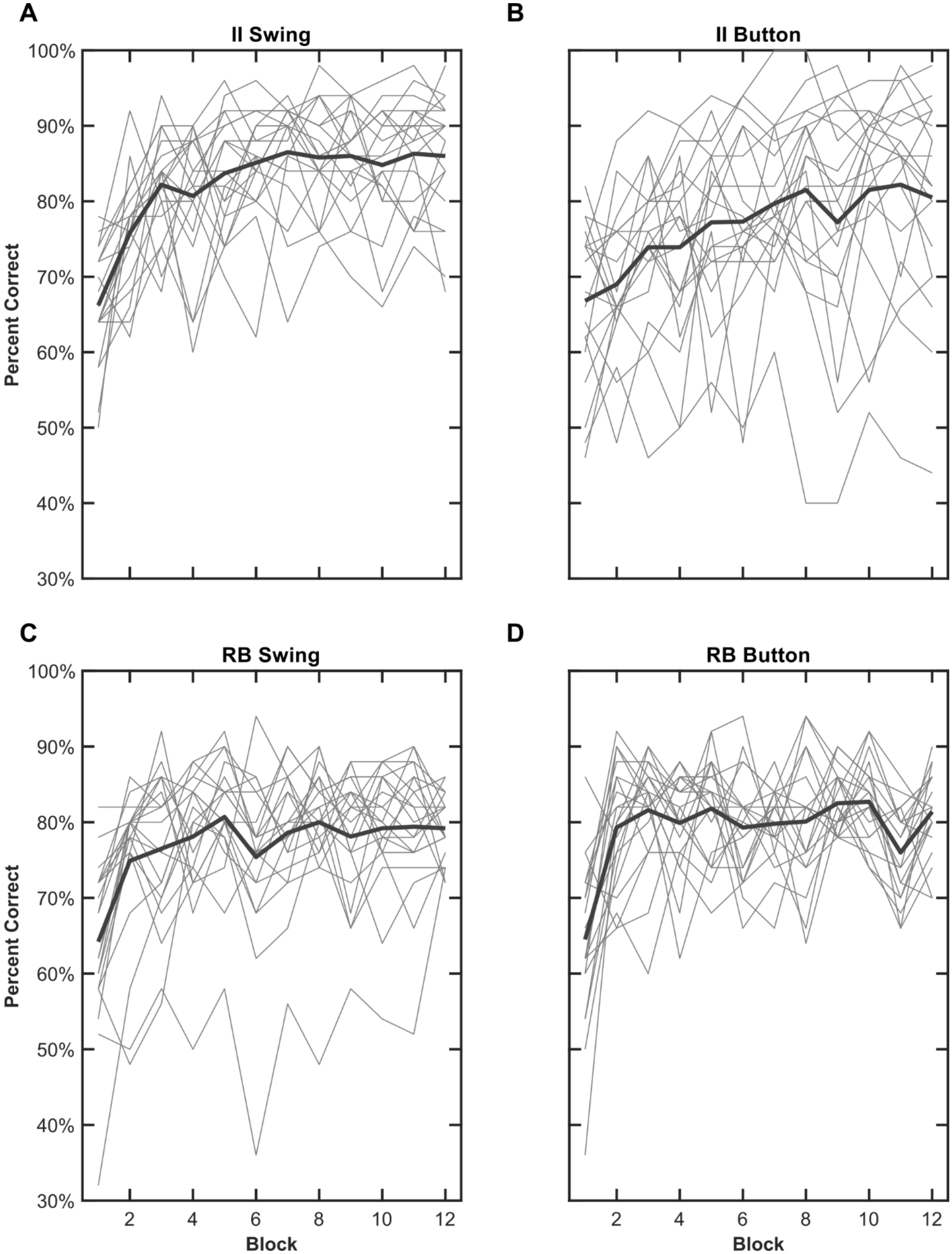

Individual (light gray) and mean (black) learning curves from Experiment 1 for the (**A**) II Swing, (**B**) II Button, (**C**) RB Swing, and (**D**) RB Button Conditions.

Fig. S2: Individual Learning Curves from Experiment 2

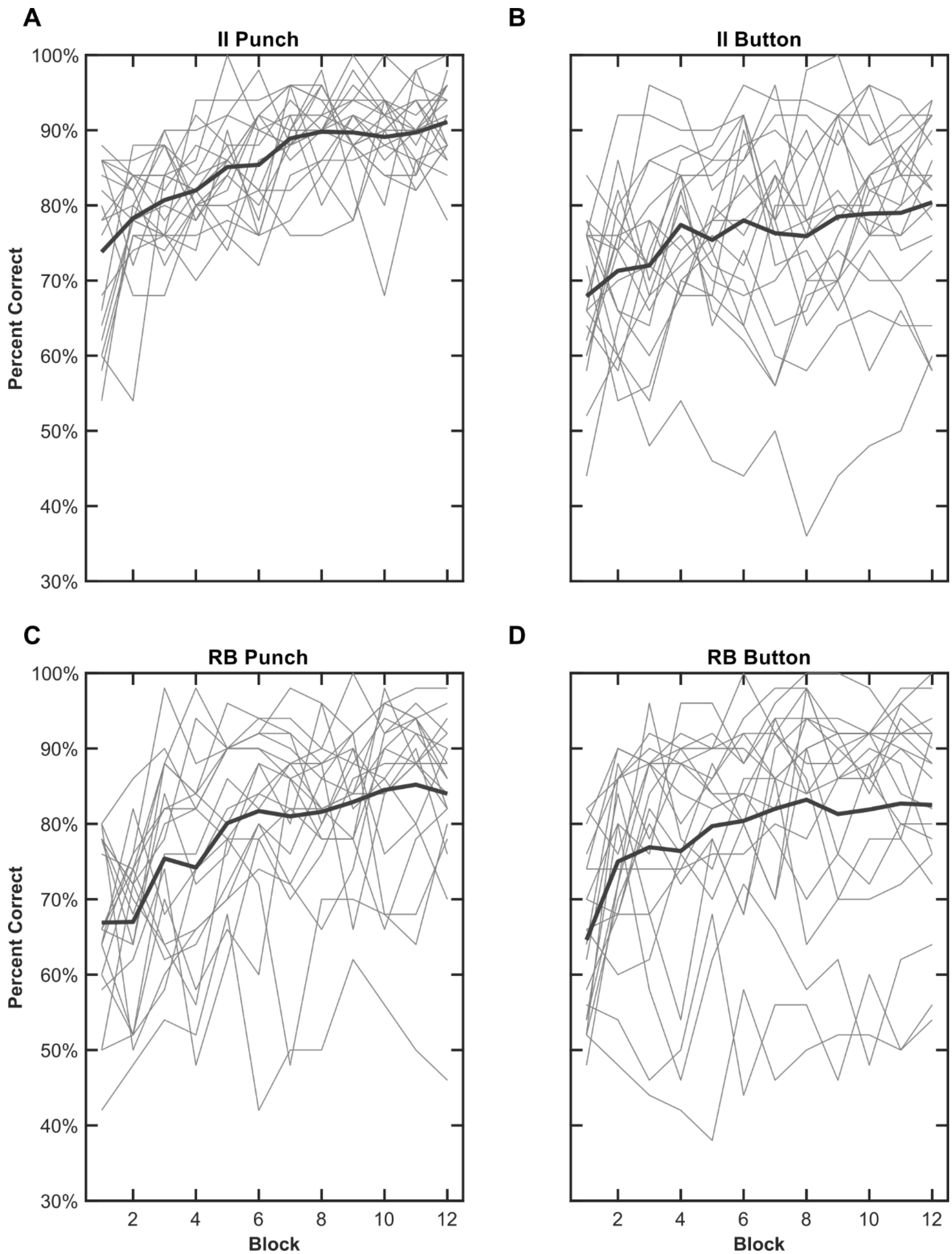

Individual (light gray) and mean (black) learning curves from Experiment 2 for the (**A**) II Punch, (**B**) II Button, (**C**) RB Punch, and (**D**) RB Button Conditions.

Table S1: Full GLMMs Experiment 1

Formula	AIC	ΔAIC	BIC	ΔBIC
PC ~ 1 + IIRB + Swing + Block + IIRB:Swing + IIRB:Block + (Block|Subject)	**5362.46**	**0**	**5406.26**	**0**
PC ~ 1 + IIRB + Swing + Block + IIRB:Swing + Swing:Block + IIRB:Block + (Block|Subject)	5363.62	1.17	5412.29	6.03
PC ~ 1 + IIRB*Swing*Block + (Block|Subject)	5365.60	3.14	5419.13	12.87
PC ~ 1 + IIRB + Swing + Block + IIRB:Block + (Block|Subject)	5368.14	5.68	5407.07	0.82
PC ~ 1 + IIRB + Swing + Block + Swing:Block + IIRB:Block + (Block|Subject)	5369.31	6.85	5413.11	6.85
PC ~ 1 + IIRB + Swing + Block + IIRB:Swing + (Block|Subject)	5375.81	13.35	5414.74	8.48
PC ~ 1 + IIRB + Swing + Block + IIRB:Swing + Swing:Block + (Block|Subject)	5377.21	14.76	5421.01	14.76
PC ~ 1 + IIRB + Swing + Block + (Block|Subject)	5381.52	19.06	5415.59	9.33
PC ~ 1 + IIRB + Swing + Block + Swing:Block + (Block|Subject)	5382.92	20.46	5421.86	15.60

Generalized Linear Mixed Models run on the full dataset (both category structures) of Experiment 1.

Table S2: GLMMs Separated by II/RB for Experiment 1

Category Structure	Formula	AIC	ΔAIC	BIC	ΔBIC
II	PC ~ 1 + Swing + Block + (Block|Subject)	**2627.6**	**0**	2652.6	0.3
II	PC ~ 1 + Swing*Block + (Block|Subject)	2629.3	1.7	2658.5	6.2
II	PC ~ 1 + Block + (Block|Subject)	2631.5	3.9	**2652.3**	**0**
RB	PC ~ 1 + Block + (Block|Subject)	**2711.5**	**0**	**2732.4**	**0**
RB	PC ~ 1 + Swing + Block + (Block|Subject)	2712.2	0.7	2737.3	4.9
RB	PC ~ 1 + Swing*Block + (Block|Subject)	2713.7	2.2	2743.0	10.6

Generalized Linear Mixed Models run separately on the Information-Integration and Rule-Based datasets of Experiment 1.

Table S3: Full GLMMs Experiment 2

Formula	AIC	ΔAIC	BIC	ΔBIC
PC ~ 1 + IIRB + Punch + Block + IIRB:Punch + Punch:Block + (Block|Subject)	**5397.5**	**0**	5441.3	2.19
PC ~ 1 + IIRB + Punch + Block + IIRB:Punch + Punch:Block + IIRB:Block + (Block|Subject)	5399.0	1.50	5447.6	8.55
PC ~ 1 + IIRB*Punch*Block + (Block|Subject)	5399.7	2.20	5453.2	14.13
PC ~ 1 + IIRB + Punch + Block + IIRB:Punch + (Block|Subject)	5400.3	2.81	5439.2	0.13
PC ~ 1 + IIRB + Punch + Block + IIRB:Punch + IIRB:Block + (Block|Subject)	5401.8	4.28	5445.6	6.47
PC ~ 1 + IIRB + Punch + Block + Punch:Block + (Block|Subject)	5402.2	4.71	5441.1	2.03
PC ~ 1 + IIRB + Punch + Block + Punch:Block + IIRB:Block + (Block|Subject)	5403.7	6.22	5447.5	8.41
PC ~ 1 + IIRB + Punch + Block + (Block|Subject)	5405.0	7.54	**5439.1**	**0**
PC ~ 1 + IIRB + Punch + Block + IIRB:Block + (Block|Subject)	5406.5	9.03	5445.4	6.35

Generalized Linear Mixed Models run on the full dataset (both category structures) of Experiment 2.

Table S4: GLMMs Separated by II/RB for Experiment 2

Category Structure	Formula	AIC	ΔAIC	BIC	ΔBIC
II	PC ~ 1 + Punch*Block + (Block|Subject)	**2607.8**	**0**	2637.0	0.2
II	PC ~ 1 + Punch + Block + (Block|Subject)	2611.7	3.9	**2636.8**	**0**
II	PC ~ 1 + Block + (Block|Subject)	2621.8	14.0	2642.7	5.9
RB	PC ~ 1 + Block + (Block|Subject)	**2788.0**	**0**	**2808.8**	**0**
RB	PC ~ 1 + Punch + Block + (Block|Subject)	2789.2	1.2	2814.3	5.5
RB	PC ~ 1 + Punch*Block + (Block|Subject)	2790.7	2.7	2819.9	11.1

Generalized Linear Mixed Models run separately on the Information-Integration and Rule-Based datasets of Experiment 2.

Table S5: GLMMs with Reaction Time Experiment 2

Formula	AIC	ΔAIC	BIC	ΔBIC	RT Estimate
PC ~ 1 + Punch*Trial + Punch*RT + Trial*RT + (RT + Trial|Subject)	**21938.1**	**0**	22043.2	17.1	-0.99386
PC ~ 1 + Punch*Trial + Punch*RT + (RT + Trial|Subject)	21940.8	2.7	22037.8	11.7	-1.11597
PC ~ 1 + Punch*Trial + Trial*RT + (RT + Trial|Subject)	21942.1	4.0	22039.2	13.0	-0.77159
PC ~ 1 + Punch*RT + Trial*RT + (RT + Trial|Subject)	21943.0	4.9	22040.0	13.9	-0.95446
PC ~ 1 + Punch + Trial*RT + (RT + Trial|Subject)	21944.3	6.3	22033.3	7.2	-0.77269
PC ~ 1 + Punch*Trial + RT + (RT + Trial|Subject)	21944.8	6.7	22033.7	7.6	-0.89363
PC ~ 1 + Punch*RT + Trial + (RT + Trial|Subject)	21945.4	7.3	22034.3	8.2	-1.08138
PC ~ 1 + Punch + Trial + RT + (RT + Trial|Subject)	21947.0	8.9	22027.9	1.7	-0.89458
PC ~ 1 + Trial*RT + (RT + Trial|Subject)	21950.8	12.7	22031.7	5.5	-0.78986
PC ~ 1 + Trial + RT + (RT + Trial|Subject)	21953.4	15.3	**22026.1**	**0**	-0.91046
PC ~ 1 + Punch*Trial + (Trial|Subject)	22394.2	456.1	22450.8	424.7	NA
PC ~ 1 + Punch + Trial + (Trial|Subject)	22398.5	460.4	22447.0	420.9	NA
PC ~ 1 + Trial + (Trial|Subject)	22408.3	470.2	22448.7	422.6	NA

Generalized Linear Mixed Models for Information-Integration categories with factor for reaction time.
